# SARS-CoV-2 Morphometry Analysis and Prediction of Real Virus Levels Based on Full Recurrent Neural Network Using TEM Images

**DOI:** 10.3390/v14112386

**Published:** 2022-10-28

**Authors:** Bakr Ahmed Taha, Yousif Al Mashhadany, Abdulmajeed H. J. Al-Jumaily, Mohd Saiful Dzulkefly Bin Zan, Norhana Arsad

**Affiliations:** 1 Department of Electrical, Electronic and Systems Engineering, Faculty of Engineering and Built Environment, Universiti Kebangsaan Malaysia, Bangi 43600, Malaysia; 2 Department of Electrical Engineering, College of Engineering, University of Anbar, Anbar 00964, Iraq; 3 Department of Computer and Communication Systems Engineering, Universiti Putra Malaysia, Serdang 43400, Malaysia

**Keywords:** SARS-CoV-2, artificial intelligence, RNN, morphometry, transmission electron microscopy

## Abstract

The SARS-CoV-2 virus is responsible for the rapid global spread of the COVID-19 disease. As a result, it is critical to understand and collect primary data on the virus, infection epidemiology, and treatment. Despite the speed with which the virus was detected, studies of its cell biology and architecture at the ultrastructural level are still in their infancy. Therefore, we investigated and analyzed the viral morphometry of SARS-CoV-2 to extract important key points of the virus’s characteristics. Then, we proposed a prediction model to identify the real virus levels based on the optimization of a full recurrent neural network (RNN) using transmission electron microscopy (TEM) images. Consequently, identification of virus levels depends on the size of the morphometry of the area (width, height, circularity, roundness, aspect ratio, and solidity). The results of our model were an error score of training network performance 3.216 × 10^−11^ at 639 epoch, regression of −1.6 × 10^−9^, momentum gain (Mu) 1 × 10^−9^, and gradient value of 9.6852 × 10^−8^, which represent a network with a high ability to predict virus levels. The fully automated system enables virologists to take a high-accuracy approach to virus diagnosis, prevention of mutations, and life cycle and improvement of diagnostic reagents and drugs, adding a point of view to the advancement of medical virology.

## 1. Introduction

In 2002–2003, an outbreak of SARS-CoV-1 in China occurred, with 8000 confirmed cases and 800 deaths. In 2012, the Middle East Respiratory Syndrome Coronavirus (MERS-CoV) was responsible for recurrent outbreaks on the Arabian Peninsula. SARS-CoV and MERS-CoV were determined to have infected civets and camels, respectively [1], before reaching humans. In April 2020, it was reported in Wuhan, China, with 1,844,683 confirmed cases and 117,021 deaths worldwide. The World Health Organization (WHO) had designated it as a pandemic [2,3,4,5]. Developing quick and reliable diagnostic techniques is crucial during the current coronavirus disease 2019 (COVID-19) pandemic, as scale-based diagnostic capability is critical to controlling outbreaks and lowering death rates. If no vaccinations or treatments can effectively stop the spread of the disease, the best method to contain it is to isolate all affected people, regardless of whether they show symptoms or not [6,7,8,9]. Patient identification and contact tracking may be completed more quickly with fast diagnostics on site. Sadly, such diagnostic tools are not currently available. Although immunogenic lateral flow tests have the advantages of speed, portability, and low cost, they are not suitable for the detection of early-stage viruses [10]. Chemical, physical, and molecular biology and morphological procedures are the most commonly used for viral analysis [11]. However, the process has advantages such as high precision and short testing time, as well as drawbacks such as secondary contamination of chemical reagents, expensive equipment, long testing time, and extended training time for skilled operators [12]. PCR-based nucleic acid amplification assays are the gold standard to confirm COVID-19 due to their high analytical precision (99%) [13,14]. However, most PCR tests are performed in centralized laboratories, leading to logistical overhead (such as sample movement and protection against deterioration) and long turnaround times (2–3 days). Furthermore, the long test periods (1–2 h) and the need for bulky equipment prevent widespread use of traditional PCR in point-of-care (POC) settings. Although new isothermal nucleic acid amplification methods have the potential to reduce test times, they are not yet as well established as traditional PCR (due to supply chain difficulties and the requirement for clinical validation) [15,16,17]. The following article discusses RNN to predict SARS-CoV-2 and other pathogenic viruses (SARS-CoV-1, MERS-CoV, Ebola, Dengue, and Influenza). It is crucial to use a predictor to determine their differences based on their genetic profiles. This study proposes the COVID Deep Predictor, an RNN-based method for detecting pathogens in a sample of unknown sequences [18]. As SARS-CoV-2 and other coronaviruses have similar genomic structures and symptoms, it may be difficult to identify this virus at an early stage. This paper presents an automated method for detecting SARS-CoV-2 cases using deep neural networks [19]. It is proposed to develop a deep bidirectional RNN that is capable of identifying SARS-CoV-2 from its viral genome sequences [20].

### 1.1. Research Contribution

Viral particles are typically too minute to observe with a conventional light microscope. Therefore, electron microscopy is commonly utilized in the field of virology. In many scenarios, such as when trying to diagnose a virus in a clinical setting or to understand how it enters and assembles its particles, morphological analysis of the virus is required. Furthermore, if a virus is propagated in cell culture, particularly if the viral genome has been altered, quality control of virus particle integrity is necessary. In addition, conventional analysis methods are laborious and time consuming. In addition, it can reduce the misjudgments of biologists to some extent, and their efficiency can be increased. The aim of this paper is to improve the accuracy of virus detection and help virologists and medical teams provide effective attention to the diagnostic process. In this paper, we proposed a fully automated mixed-attention algorithms based on an optimized recurrent neural network (RNN) that helps advance medical virology by enabling virologists with a high-accuracy approach to the identification and detection of the SARS-CoV-2 virus. Thus, it is helpful to assist professionals in their laborious work by combining approaches of the attention mechanism for recognition and categorization.

### 1.2. Research Background 

The viruses are divided into two categories: First are the types of viruses that are helpful. Some benefits include that they can break down things and provide nutrients to plants, they can decompose organic material, improve the quality of freshwater, and they can help fix nitrogen and provide nourishment to humans [21]. Second, there are harmful types that cause diseases and death, such as the new coronavirus disease 2019 (COVID-19), which constitutes a worldwide public health emergency [22,23]. Consequently, the study of viruses is critical for associated research and applications such as pollutant monitoring and environmental management and medical diagnostics, agriculture, and food production [24,25,26]. Referring to the literature to justify virus analysis methods can be divided into four broad categories: chemical (analysis of chemical components), physical (spectrum analysis), molecular, biological (DNA and RNA analysis), and morphological (structure of organisms) [27,28]. Despite its great degree of precision, the chemical approach frequently leads to secondary contamination of chemical reagents. Because it involves expensive equipment, a physical technique is also very accurate [29,30]. However, the study of a genome sequence differentiates viruses using the molecular biological technique. This approach requires a large amount of costly equipment. The use of artificial intelligence (AI) has made significant advances in recent years. It has been demonstrated that artificial intelligence (AI) and deep learning are increasingly being utilized in biomedical sciences, for example, for the recognition of images, restorations upon low light sampling, and object segmentation [31,32]. Human adenovirus movements inside host cells are classified using a unique supervised trajectory segmentation algorithm [33]. This presents an efficient and machine-independent method for analyzing CT-based COVID-19 images for segmentation and quantification [34]. 

As a result of its remarkable performance in a wide range of image analysis and processing domains [35], such as face recognition [36], autonomous driving [36], integration with the laser devices [37,38], optical properties [39,40,41], and diagnosis of diseases [8,17], image data can be quickly analyzed using AI technology, which can perform tedious and time-consuming tasks. An automated method aims to develop medical virology by providing virologists with a high-accuracy approach to recognizing viruses that helps the diagnosis process [42]. Several AI classification techniques have been used for this problem. Segmentation represents the main challenge in medical imaging due to its extraordinary imaging performance. Combining AI techniques for recognition and classification is possible, freeing specialists from laborious work. In AI applications, fine-grained image classification is a different task, since the shape and geometric characteristics of fine-grained categories are very similar; therefore, it is important to identify fine-grained images through differences between key parts. This insight was also applied to virus classification [43,44]. The physical, medicinal, and biological sciences benefit greatly from fluorescence-microscopy-based volumetric material imaging. This study used a recurrent neural network (RNN) to perform volumetric fluorescence microscopy [45]. In addition, biometric authentication and anomaly detection were performed using recurrent neural networks [46]. 

## 2. The Role of AI in Diagnosis and Prediction

**Figure 1:**
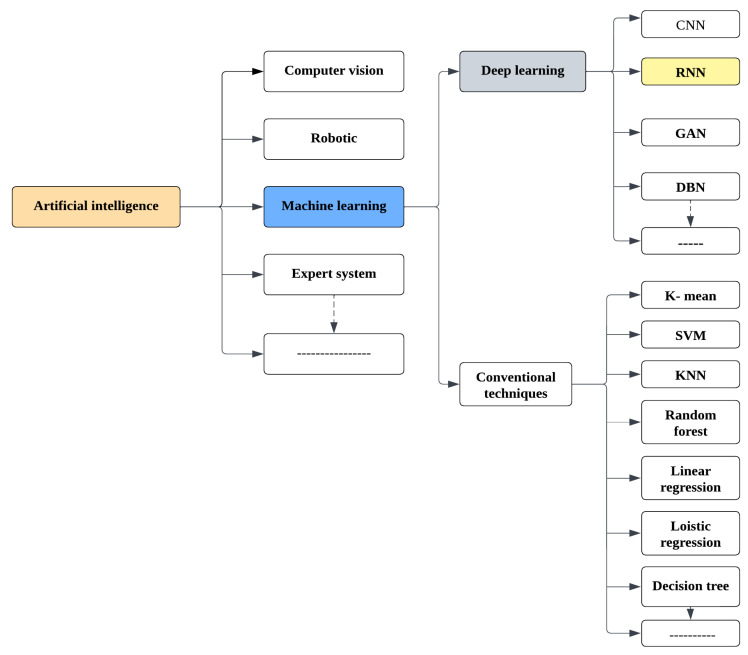
Flowchart illustrating AI techniques for virus detection and classification.

Artificial intelligence (AI) describes approaches that allow a machine to replicate or surpass human intelligence, primarily in cognitive capacities. Machine learning (ML), computer vision, and natural language processing are the main subdomains of AI [47,48]. ML technology, for example, has been frequently applied in microorganisms such as in environmental microorganism (EM) segmentation. ML may be divided into two categories: traditional approaches and artificial neural networks (ANNs). Support vector machine (SVM), k-nearest neighbor (KNN), random forest (RF), and other algorithms have previously been used to analyze viruses. Additionally, with the help of artificial intelligence, even the most time-consuming tasks can be completed in a fraction of the time. It follows that microorganism image analysis (MIA) could benefit from AI [49]. As a result, AI is capable of objective analysis in MIA and avoids subjective differences that may arise from manual accounting. Biologists’ misjudgments can be decreased, and their efficiency can be increased. ANNs are a critical component of AI technology that was first inspired by the structure and function of a biological neuron. A lack of computing power, training difficulties, and widespread use of support vector machines (SVM) stifled the early stages of artificial neural network (ANN) research. Convolutional neural networks (CNNs) have shown an overwhelming advantage in image recognition, and ANNs have been re-evaluated and evolved fast due to this. Because ANNs can discover significant patterns from large datasets, they are commonly used in MIA investigations [7]. Moreover, this paper study has demonstrated the feasibility of using a CNN to automatically evaluate Gram stains from blood culture. The authors reported their results in 2018. This work sheds light on many critical prospective AI applications in clinical microbiology. The authors used a CNN, which is particularly good at classifying images. The problem is that training such a sophisticated network is computationally costly and therefore requires specialized equipment [50]. Figure 1 shows the architecture of AI methods. 

## 3. Artificial Neural Network Algorithms

There has been considerable interest in artificial neural networks (ANNs) as a method for simulating complex real-world problems computationally. Analyzing non-linear data with ANNs is a statistical method. Non-linear functions are predetermined at the beginning of the process. Output data are mapped to input data [51,52]. The input values (variables) are represented by $x_{i},\;I=1,\ldots ,n,$ where n is the number of variables, and the first layer creates M linear combinations of $x_{i}$, from which the intermediate activation variables $a_{j}\;$ are generated. Each hidden layer has an associated variable, denoted as $a_{j}$, and $w_{ji}$ denotes the elements of the first layer weight matrix, and $w_{j0,}$ are the bias variables linked with the hidden unit’s *j*, as shown in Equation (1).

(1) $$a_{j}=\sum_{i=1}^{n}\left(w_{ji}\;x_{i}\right)+w_{j0},\;j=1,\ldots ,M\;$$

### 3.1. Recurrent Neural Network (RNN) 

**Figure 2:**
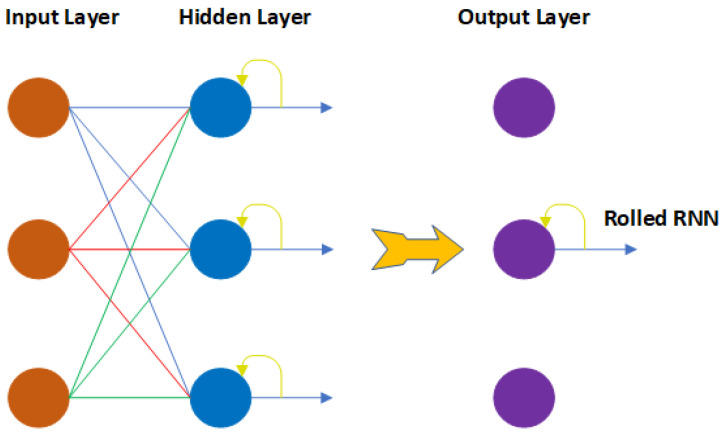
Conceptual representation of an RNN.

In computing, recurrent neural networks (RNNs) represent a diverse collection of models that attempt to mimic the functions of the human brain. Multiple “conceptual neurons,” or “processing elements,” in an RNN are linked together through synaptic connections. An RNN’s ability to generate self-sustained temporal activation dynamics along its recurrent connection pathways, even without input, sets it apart from the more general feedforward neural networks. Since feedforward networks are functions, this mathematically demonstrates that an RNN is a dynamic system. In response to an input signal, an RNN maintains, in its internal state, a nonlinear transformation of the input history. It allows the RNN to interpret temporal context information and gives the network a dynamic memory [28]. Figure 2 represents a schematic diagram of the recurrent neural network. Generally, RNNs have three primary parts to their architecture: input, hidden neuron, and activation function, which can be defined as Equation (2).

(2) $$h_{t}=\tanh\left(U\cdot X_{t}+W\cdot h_{t-1}\right)\;$$

Here, $X_{t}$ is the input at time t, $h_{t}$ is the hidden neuron at time, U denotes the weight of the hidden layer, and W is the transition weights of the hidden layer. The input and previous hidden states are merged to yield information as the current and past inputs pass through the tanh function. The result is a new hidden state that acts as a memory for the neural network, storing data from the original network [53].

### 3.2. General Regression Neural Network (GRNN) 

**Figure 3:**
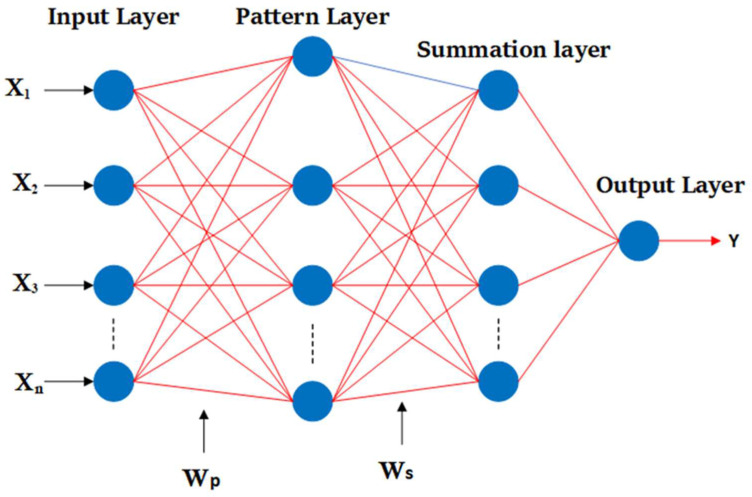
Conceptual representation of a GRNN [56].

The generalized regression neural network (GRNN) is a well-known statistical technique for predicting functions and is dependent on nonlinear regression [54]. The architecture is a neural network architecture that can handle any function approximation issue in estimating a probability distribution function. When adequate data are given, the GRNN is a universal approximator for smooth operations [55], allowing it to solve any function approximation and estimate any continuous variable issue. This architecture is a highly parallelized one-pass learning method. The approach enables smooth transitions from one observed value to another, even with sparse data in a multidimensional measurement space. Figure 3 shows the four layers of the GRNN (input, pattern, summation, and output); each unit in the pattern layers represents an input pattern used for training. The distance between input and the stored patterns is calculated by the unit’s output, which is connected by the pattern layer weights ($W_{P}$). Each unit in the pattern layer communicates with a neuron in the summation layer through weights ($W_{S}$). The S-summation neuron computes the sum of the weighted output of the pattern layer. This results in the projected value for an unknown input vector x, as shown in Equation (3).

(3) $$Y_{i}=\frac{\sum_{i=1}^{n}y_{i}\;.exp\left[-D\;\left(x,\;x_{i}\right)\right]}{\sum_{i=1}^{n}\;.exp\left[-D\;\left(x,\;x_{i}\right)\right]}\;$$

Here, *n* is the total number of training patterns, $y_{i}$ is the weight connection between the ith neuron, and D is the Gaussian function, as defined in Equation (4):

(4) $$D\left(x,\;x_{i}\right)=\sum_{k=1}^{m}{\left(\frac{x_{k}-x_{ik}}{\sigma }\right)}^{2}$$

where *m* is the number element of the input vector, $x_{k}$ and $x_{ik}$ are the jth element of $x,\;x_{i}$, and σ. The spread (or width) impacts the generalization performance of the GRNN.

## 4. Methodology 

**Figure 4:**
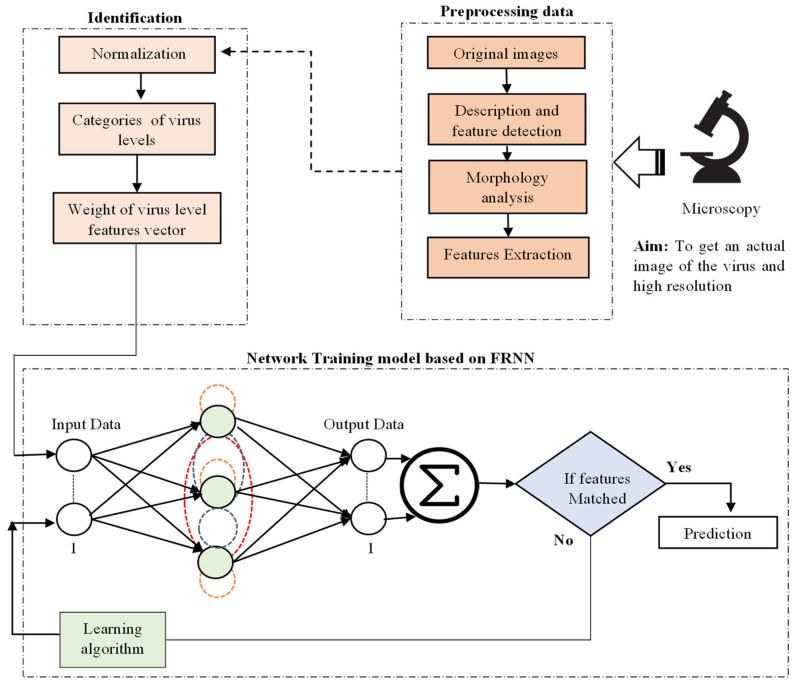
The SARS-CoV-2 prediction model illustrates the virus levels using the full recurrent neural network (FRNN) algorithm.

Researchers have been trying to figure out the structure of viruses since the late 19th century after realizing that viruses were the underlying cause of disease. Electron microscopy (EM) has the potential to provide direct images of viruses for diagnosis and study, which has a high resolution power that allows investigations at the nanoscale [57]. Electron microscopy (EM) is crucial to studying viral replication and finding new infections. It is essential to understand the biology of viruses and the causes of viral diseases to develop effective strategies for disease prevention, accurate diagnostics, and the management of viral outbreaks [58]. In Figure 4, we propose a new prediction model of the levels of the SARS-CoV-2 virus based on an FRNN from TEM images. Adjusting the final output of the training system by optimizing the tuning of the RNN utilizing the characteristics of responses in the full recurrent neural network (FRNN). The main contribution in this article is tuning the RNN, according to the desired output weight of the hidden layer and its bias adjustment, to obtain the suitable tuning factor with the output. This has been implemented using the MATLAB 2020 software package. The methodology can help identify the life cycle of viruses and mutations, estimate disease progress, and reduce laborious or time-consuming work.

### 4.1. Data Availability and Computation

Patients with COVID-19 isolated in Italy/INMI were divided into nine groups in the dataset acquired from the Zenodo website. The SARS-CoV-2 dataset consists of 519 TEM images in Vero cell cultures via plastic discs that are so thin they are barely visible (60–70 nm). Additionally, a 16-bit image viewer was required to view the images. All image files have been calibrated to fit so that they can be opened in ImageJ or Fiji using the Bio-formats importer [59].

### 4.2. Measurement and Extraction Features 

**Figure 5:**
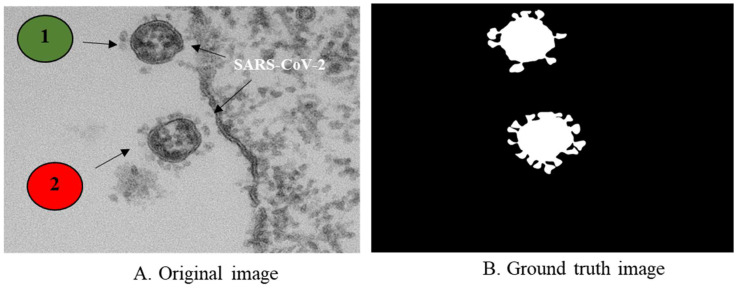
Illustration of morphometry of SARS-CoV-2 virus image.

Many investigations have found that the virus has a spherical shape with a diameter between 60 and 140 nm, giving the virus the appearance of a solar corona under an electron microscope. Virus particles, free and encapsulated in membrane-bound vesicles, were found in ultrathin sections of the human airway epithelium [60,61,62]. The area of virus particles was estimated based on Fiji software using freehand selection tools. It takes the type of selection made and provides figures of the area of the results, line lengths, angles, or point values [63]. We calculated the SARS-CoV-2 particle area data for a selected subregion specified by the selection of free-hand tools in the toolbar. Then, we examined the particles using the analysis command and measured the area of the virus in binary images. The analysis was carried out based on the region selection provided. It operates by scanning the selection until it discovers the edge of the virus shape. It then outlines using the Wand tool with the measure (m) command, fills it to make it invisible, and then starts scanning until it reaches the end of the selection. Finally, the extraction of features of the SARS-CoV-2 particle includes the area, circularity, roundness, aspect ratio, intensity, width, height, and solidity and was carried out as shown in the Supplementary File. Analysis of the geometric features of the size and shape of the SARA-CoV-2 virus may be quantified by morphometry, as shown in Figure 5.

**Figure 6:**
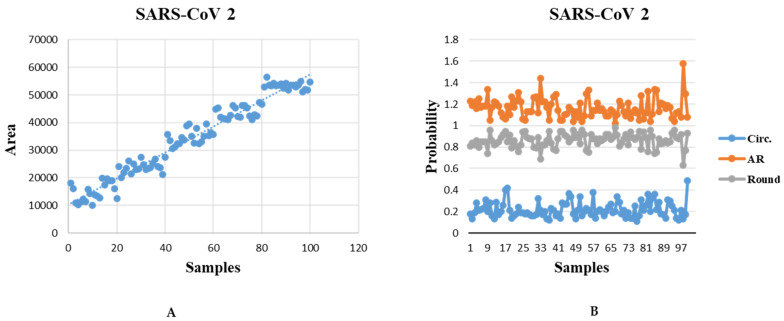
Illustration of the morphometry analysis of SARS-CoV-2: (**A**). density of area; (**B**). distribution of probability (circularity, AR, and roundness).

Figure 6 describes the shape analysis of the SARS-CoV-2 virus to estimate morphological feature metrics. First, in Figure 6A, the results present the distribution of virus area within samples, which includes a change in the size of the virus area. Second, Figure 6B shows the distribution of probability (circularity, roundness, and aspect ratio) of the samples. A perfect circle would have a circularity of 1; hence, this term describes the condition where the process is as close to perfect as possible. The further the value moves toward 0.0, the more stretched out the shape appears to be. The circularity results in the blue-colored line are under 1, meaning that the virus is not circular; the data analysis procedure generates the virus particle’s fitting ellipses’ aspect ratio in the orange color line, and the gray color line is the density of roundness of the virus.

## 5. Results

**Figure 7:**
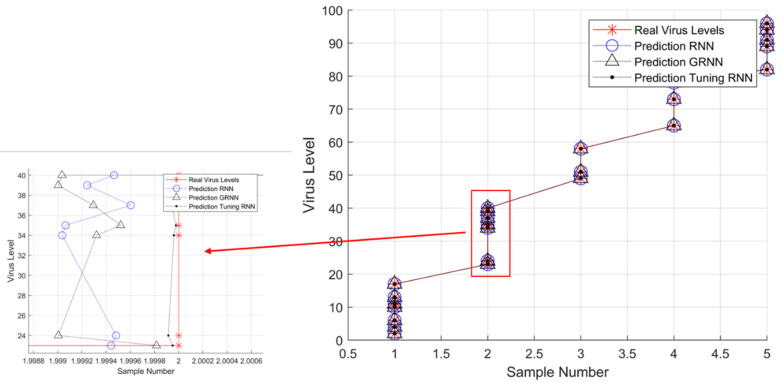
The prediction of virus levels is based on RNN, GRNN, and tuning RNN in comparison to the real data.

TEM is a valuable technology that directly identifies virus species; it reveals species-specific morphological details and quantifies all viral particles, infected or not. However, despite its usefulness, identifying between virus subtypes has no practical application due to their overlap. On the other hand, TEM may be used in other situations. Furthermore, we can potentially use it in the study of antiviral medications. To further investigate viral pathogenesis and determine the cellular target of invasion, TEM may help observe the mechanism by which antiviral drugs inhibit viral invasion of cells [64]. Different viruses have emerged over time due to divergent evolution in their respective hosts. Therefore, we need to develop an automatic prediction model for the characterization of the SARS-CoV-2 virus that can interpret interactions between viruses and their hosts, such as viral entry, replication, mutation and escape, virus levels, and virus structure, which are of considerable interest. Although various models capture individual virus families, their general properties are accessible. However, preparation procedures for investigating different viruses are more or less similar [65]. In Figure 7, we tested the morphological characteristics of the SARS-CoV-2 virus particles using virus TEM images to predict virus levels based on the RNN and GRNN. The results indicated that the prediction of real virus levels based on the tuning of the RNN was the best comparison between RNN and GRNN.

**Figure 8:**
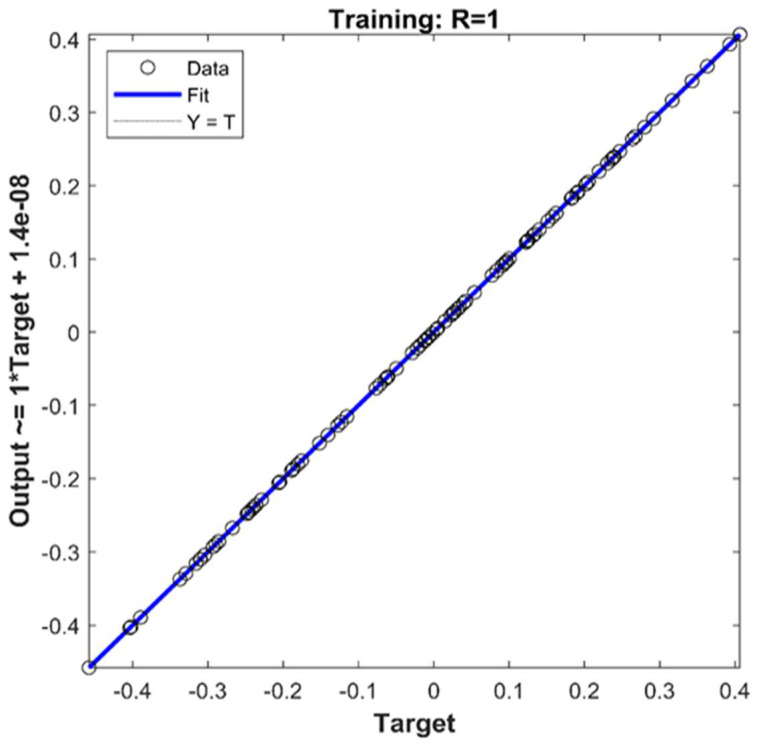
The regression model training analysis’s prediction error.

The relationship between two variables is determined using linear regression. There are two factors at play here: a dependent variable that acts as a brake on the transformation and an independent variable that acts as a catalyst. This method aims to focus on the link between the two variables rather than calculating their dependence. The activation function in each layer of an ANN allows it to understand the intricate connection between the characteristics and the target. Figure 8 represents the R2 linear regression analysis of neural network training to demonstrate the learning of the linear relationship between output and target. The error in the regression was −1.6 × 10^−9^, and the actual turbidity level is on the Y axis, with the regression estimate on the X axis. Moreover, in this paper, the results demonstrate the accuracy of the virus level prediction model.

**Figure 9:**
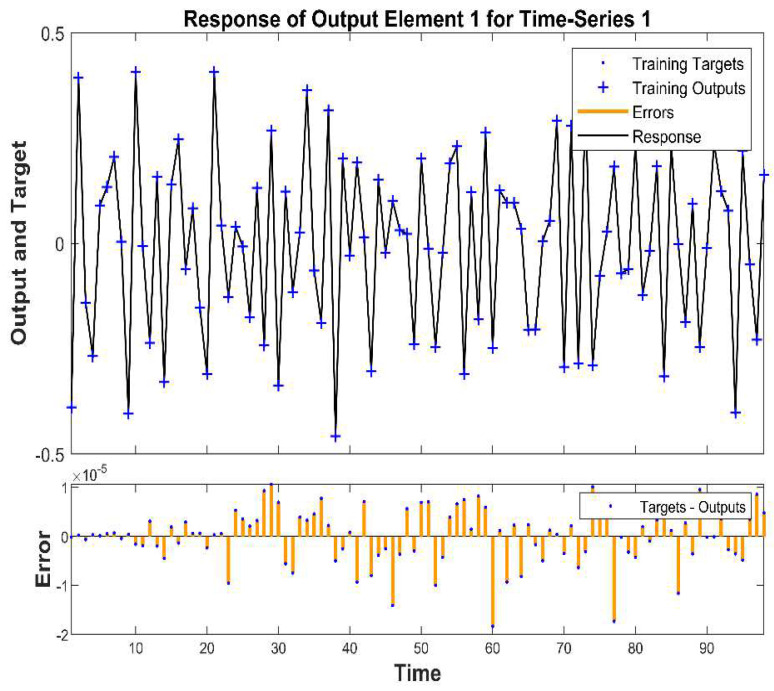
The responses of the target and output time series are based on training process.

The plot target and output time-series data of the network training response are used to show the results of virus predictions in the training stages. The time-series response is where the output is the prediction, and the goal refers to accurate data in the three stages of training and the error related to the data, as shown in Figure 9. Plots were used to compare the observed (target) values with the model’s predicted (output) values over time to evaluate the model’s performance and anticipate how well it would fare in the future concerning the forecast and the incidence of the virus at various levels. The process errors were also analyzed by plotting them against time. The model can reflect and mimic the desired output and does an excellent job of capturing the general trend at various levels of incidence. Furthermore, the estimation errors versus time range were −2 to 1 × 10^−5^, and the output and target response were between −0.5 and 0.5, which concluded that this was the best model to use.

**Figure 10:**
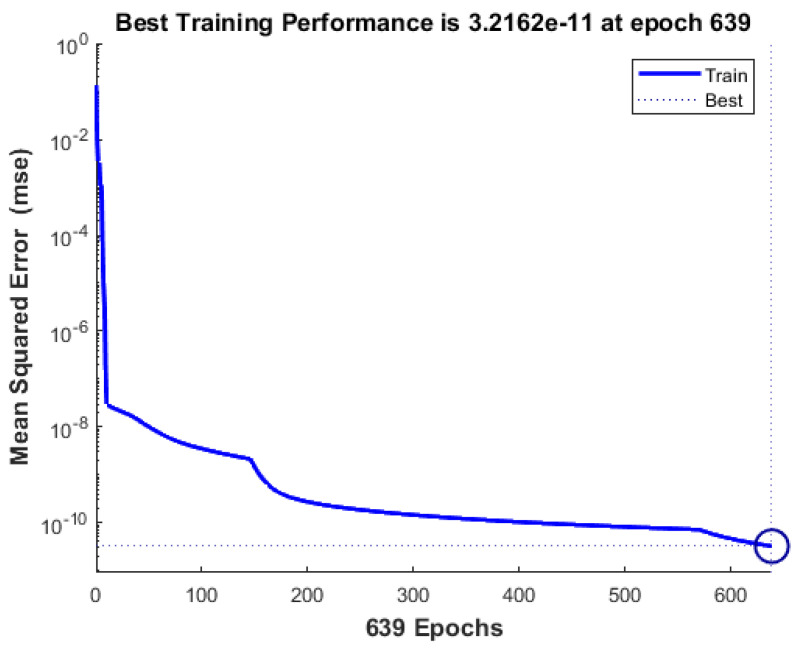
MSE performance training for the SARS-CoV-2 virus.

Figure 10 represents a performance assessment plot that shows the progress of the training. There was no evidence of overfitting. Furthermore, it indicated a similar tendency in the behavior of the training curves, which is expected given that the raw data were normalized before use. A lower mean square error determines the training accuracy of a network. An error score of 3.216 × 10^−11^ at epoch 639 indicates a network with a high ability to anticipate virus levels.

**Figure 11:**
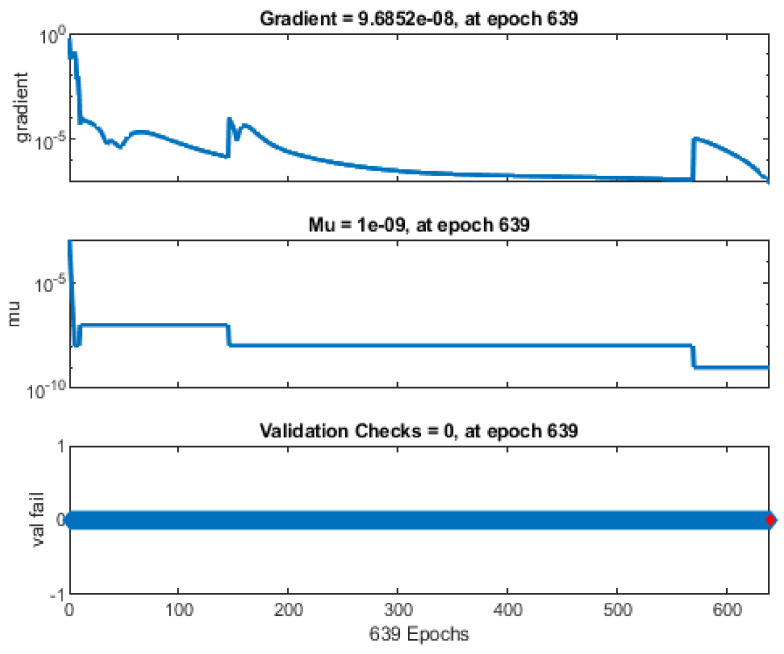
Illustration of the gradient function, the training gain (Mu), and the validation check.

In the backpropagation process after a batch of data training, the neural networks estimate the error contribution of each neuron. Technically, the neural network computes the loss function’s gradient to explain each chosen neuron’s error contribution. The gradient value of 9.6852 × 10^−8^, as shown in Figure 11, demonstrates that the error contribution of each selected neuron is small. Momentum gain (Mu) is a control parameter for the neural network training process and was 1 × 10^−9^ at epoch 639. In addition, the following figure shows the training state, including the gradient function, the training gain (Mu), and the validation check.

## 6. Discussion

Studies have shown that understanding and interpretation of the characteristics of SARS-CoV-2 is important, as follows: The structure of the spike portion enables the development of a new class of drugs for the treatment of COVID-19 [66,67]. Analysis of viral structure, surface proteins, and genome sequence has emphasized the development of diagnostic studies of the new SARS-CoV-2 virus and rapidly evolving diagnostic technologies, vaccination trials, and cell entry inhibitor medications [68]. SARS-CoV-2 has been known to mutate in many countries, and this variation can change the shape and spatial distribution of S spikes. Thus, to improve the microscopic images of new viral strains, DL algorithms are easily adapted with simple global modifications [69]. Nevertheless, computer vision has had an extraordinary influence in a wide range of fields of research and in the field of medicine, especially in bioimages, and because of their non-invasive character, the application of these techniques is already necessary. However, many scientists seek to solve it with artificial intelligence methods without considering the benefits and drawbacks of the technique [70]. The researchers discovered that the COVID Deep Predictor could recognize virus classes from genomic sequences supplied by various deadly viruses using the RNN approach. The results presented precision for test datasets in the range of 99.51 and 99.94% [18]. Since viruses are often too small to be examined using a light microscope, electron microscopy is a method that is widely used in virology. It is essential to analyze viral morphology in many situations, including identifying a virus in a specific clinical case and exploring how it enters and assembles. Moreover, quality monitoring of viral particle integrity is necessary whenever a virus is propagated. In order to extract vital critical points of the characteristics of the SARS-CoV-2 virus, we investigated and analyzed its viral morphometry. We then developed a prediction model based on optimizing a full recurrent neural network (RNN) using transmission electron microscopy (TEM) images to identify real virus levels. Thus, detecting virus levels depends on the morphometry of the area, such as width, height, circularity, roundness, aspect ratio, and solidity. Our work used FRNN feedback properties to adjust the training system’s final output during the RNN process’s tuning. The main contribution of this article is the tuning of RNN based on the desired outcome. Hence, it is by adjusting the weight of the hidden layer and its bias that the preferred tuning factor can be obtained.

## 7. Conclusions

The geometric properties of coronaviruses are highly similar; consequently, it is imperative that fine-grained images be differentiated between critical portions so that they can be identified by their geometric properties. Additionally, the results of this study may contribute to a better classification of viruses based on the information obtained from this study. Furthermore, to improve the deep neural network’s virus detection capabilities, we suggest a fully automated attention network to help with fine-grained classification. Traditional approaches to analysis are tedious and time consuming. In addition, the characterization of the SARS-CoV-2 particles is essential, and maybe a reference guide is needed to test various cell types and infection schemes. The particle size is most likely dependent on the cell’s type of viral generation and the specific infection conditions, such as the number of viruses infecting a cell. In summary, we provide a new viewpoint to assist medical teams and virologists by providing a high-accuracy technique to recognize viruses and prevent virus mutations.

## Data Availability

Datasets are accessible in the Zenodo data repository: https://zenodo.org; Dataset 03: https://doi.org/10.5281/zenodo.3985110; Dataset 07: https://doi.org/10.5281/zenodo.3986580; Dataset 08: https://doi.org/10.5281/zenodo.4275703; Dataset 09: https://doi.org/10.5281/zenodo.42757; Dataset 10: https://doi.org/10.5281/zenodo.4275735; Dataset 12: https://doi.org/10.5281/zenodo.4275769.

## Supplementary Materials

The following supporting information can be downloaded at: https://www.mdpi.com/article/10.3390/v14112386/s1, Table S1: features of SARSCOV2.
