# Supplementary material for: SARS-CoV-2 Morphometry Analysis and Prediction of Real Virus Levels Based on Full Recurrent Neural Network Using TEM Images
Source: Viruses. 2022 Oct 28;14(11):2386. doi: 10.3390/v14112386 (PMC9698148; doi:10.3390/v14112386)
Supplement: Supplementary file 1 [file viruses-14-02386-s001.zip › viruses-1941334-supplementary Table S1.pdf]

Sample	Area	Width	Height	Circ.	AR	Round	Solidity	Viruses
1	18102	197	199	0.18	1.23	0.81	0.74	1
2	16057	259	242	0.13	1.19	0.84	0.68	1
3	10896	194	183	0.19	1.22	0.82	0.76	1
4	10264	155	191	0.28	1.16	0.86	0.87	1
5	11075	184	205	0.21	1.25	0.8	0.82	1
6	12182	200	193	0.22	1.17	0.85	0.77	1
7	11388	204	183	0.23	1.18	0.85	0.74	1
8	15800	227	204	0.31	1.18	0.85	0.81	1
9	14397	205	201	0.2	1.34	0.74	0.76	1
10	10139	184	176	0.28	1.05	0.96	0.79	1
11	13948	212	198	0.16	1.17	0.86	0.78	1
12	13444	217	180	0.13	1.22	0.82	0.78	1
13	12695	196	185	0.29	1.21	0.83	0.79	1
14	19889	200	173	0.17	1.18	0.85	0.76	1
15	17483	165	178	0.2	1.12	0.89	0.75	1
16	19740	213	185	0.26	1.08	0.92	0.77	1
17	18973	179	193	0.4	1.06	0.95	0.79	1
18	19007	162	182	0.42	1.18	0.85	0.86	1
19	16085	180	194	0.21	1.1	0.91	0.79	1
20	12417	214	193	0.14	1.27	0.79	0.75	1
21	24192	212	183	0.16	1.17	0.86	0.77	2
22	20102	197	199	0.18	1.23	0.81	0.74	2
23	21842	241	175	0.24	1.31	0.76	0.72	2
24	23369	219	185	0.19	1.22	0.82	0.76	2
25	26038	217	197	0.19	1.06	0.94	0.75	2
26	21331	189	198	0.18	1.05	0.95	0.8	2
27	25001	211	204	0.19	1.13	0.89	0.75	2
28	23044	210	186	0.17	1.13	0.88	0.77	2
29	23216	197	190	0.16	1.13	0.88	0.78	2
30	27394	193	231	0.16	1.26	0.8	0.81	2
31	24711	196	236	0.17	1.27	0.79	0.77	2
32	22899	193	201	0.32	1.12	0.89	0.82	2
33	23492	195	224	0.21	1.44	0.69	0.75	2
34	23759	246	210	0.17	1.22	0.82	0.72	2
35	25300	209	221	0.2	1.22	0.82	0.75	2
36	26856	213	241	0.13	1.17	0.86	0.76	2
37	24187	224	213	0.12	1.05	0.95	0.67	2
38	23567	206	196	0.23	1.2	0.84	0.8	2
39	21303	245	182	0.21	1.27	0.78	0.73	2
40	27495	201	232	0.16	1.29	0.77	0.76	2
41	35695	212	190	0.18	1.14	0.88	0.83	3
42	33599	214	192	0.14	1.05	0.95	0.74	3
43	30481	193	183	0.28	1.05	0.95	0.81	3
44	31268	199	172	0.27	1.1	0.91	0.8	3
45	32461	211	188	0.27	1.11	0.9	0.73	3
46	32290	195	175	0.37	1.17	0.85	0.83	3
47	34635	196	166	0.34	1.15	0.87	0.81	3

48	33637	201	198	0.18	1.04	0.96	0.78	3
49	38821	268	245	0.13	1.13	0.89	0.78	3
50	39589	260	258	0.21	1.08	0.92	0.8	3
51	35030	257	238	0.34	1.21	0.83	0.84	3
52	32657	237	249	0.16	1.04	0.96	0.78	3
53	37946	303	260	0.2	1.09	0.92	0.67	3
54	32325	269	193	0.23	1.3	0.77	0.79	3
55	33067	206	192	0.21	1.33	0.75	0.81	3
56	35051	213	210	0.17	1.09	0.92	0.76	3
57	39404	197	225	0.38	1.14	0.88	0.88	3
58	35336	197	210	0.14	1.14	0.88	0.78	3
59	36309	190	193	0.2	1.21	0.83	0.84	3
60	35663	220	205	0.22	1.14	0.88	0.77	3
61	44788	207	196	0.2	1.16	0.86	0.78	4
62	45304	235	220	0.16	1.13	0.88	0.6	4
63	41877	190	206	0.2	1.09	0.92	0.77	4
64	41425	192	188	0.24	1.09	0.92	0.79	4
65	41373	183	220	0.27	1.15	0.87	0.73	4
66	41108	192	191	0.19	1.13	0.89	0.73	4
67	42544	219	192	0.2	1.02	0.98	0.78	4
68	46235	185	183	0.34	1.1	0.91	0.82	4
69	45216	208	211	0.29	1.23	0.81	0.75	4
70	42227	227	177	0.18	1.19	0.84	0.8	4
71	41863	193	189	0.21	1.13	0.88	0.81	4
72	46173	213	208	0.14	1.09	0.92	0.65	4
73	46125	195	164	0.19	1.21	0.82	0.82	4
74	45333	287	273	0.14	1.07	0.94	0.77	4
75	42494	298	258	0.14	1.12	0.89	0.77	4
76	40980	263	256	0.25	1.15	0.87	0.79	4
77	42775	288	265	0.11	1.12	0.89	0.75	4
78	42351	265	264	0.16	1.05	0.95	0.81	4
79	47264	297	239	0.31	1.28	0.78	0.8	4
80	46710	197	200	0.21	1.06	0.94	0.75	4
81	52831	196	193	0.26	1.12	0.89	0.78	5
82	56372	245	186	0.36	1.32	0.76	0.72	5
83	53485	220	212	0.2	1.04	0.96	0.79	5
84	53323	195	197	0.33	1.11	0.9	0.83	5
85	54255	196	179	0.36	1.34	0.74	0.86	5
86	53431	231	188	0.22	1.33	0.75	0.75	5
87	53472	212	172	0.29	1.13	0.88	0.81	5
88	54091	223	207	0.18	1.21	0.83	0.75	5
89	52509	210	214	0.17	1.2	0.84	0.74	5
90	54344	200	205	0.14	1.16	0.86	0.79	5
91	51845	193	189	0.31	1.19	0.84	0.83	5
92	53571	176	199	0.3	1.17	0.85	0.86	5
93	53637	186	180	0.25	1.07	0.93	0.79	5
94	52944	207	215	0.21	1.04	0.96	0.73	5
95	53484	261	219	0.14	1.12	0.89	0.64	5

96	54872	241	220	0.12	1.13	0.88	0.66	5
97	51189	181	191	0.21	1.08	0.92	0.81	5
98	51970	258	179	0.13	1.58	0.63	0.63	5
99	51757	233	181	0.17	1.3	0.77	0.76	5
100	54664	305	280	0.49	1.08	0.93	0.88	5

Samples	
Dataset_02_SARS-CoV-2_066	1
Dataset_02_SARS-CoV-2_089	4
Dataset_02_SARS-CoV-2_108	6
Dataset_02_SARS-CoV-2_108	7
Dataset_03_SARS-CoV-2_008	8
Dataset_03_SARS-CoV-2_008	9
Dataset_03_SARS-CoV-2_031	12
Dataset_03_SARS-CoV-2_031	14
Dataset_03_SARS-CoV-2_031	15
Dataset_03_SARS-CoV-2_052	16
Dataset_03_SARS-CoV-2_052	17
Dataset_03_SARS-CoV-2_052	18
Dataset_03_SARS-CoV-2_052	19
Dataset_03_SARS-CoV-2_008	10
Dataset_03_SARS-CoV-2_009	11
Dataset_10_SARS-CoV-2_062	14
Dataset_09_SARS-CoV-2_029	19
Dataset_08_SARS-CoV-2_049	22
Dataset_08_SARS-CoV-2_029	6
Dataset_08_SARS-CoV-2_006	9
Dataset_03_SARS-CoV-2_092	43
Dataset_02_SARS-CoV-2_066	38
Dataset_03_SARS-CoV-2_091	39
Dataset_03_SARS-CoV-2_091	40
Dataset_03_SARS-CoV-2_091	41
Dataset_03_SARS-CoV-2_091	20
Dataset_03_SARS-CoV-2_091	22
Dataset_03_SARS-CoV-2_107	28
Dataset_03_SARS-CoV-2_107	29
Dataset_10_SARS-CoV-2_063	2
Dataset_10_SARS-CoV-2_063	3
Dataset_10_SARS-CoV-2_063	4
Dataset_10_SARS-CoV-2_063	5
Dataset_10_SARS-CoV-2_062	6
Dataset_10_SARS-CoV-2_062	7
Dataset_10_SARS-CoV-2_062	8
Dataset_10_SARS-CoV-2_062	9
Dataset_10_SARS-CoV-2_062	10
Dataset_10_SARS-CoV-2_062	11
Dataset_10_SARS-CoV-2_062	12
Dataset_02_SARS-CoV-2_088	2
Dataset_02_SARS-CoV-2_089	3
Dataset_03_SARS-CoV-2_092	23
Dataset_03_SARS-CoV-2_092	24
Dataset_03_SARS-CoV-2_092	25
Dataset_03_SARS-CoV-2_092	26
Dataset_03_SARS-CoV-2_092	27

Dataset_03_SARS-CoV-2_092	42
Dataset_03_SARS-CoV-2_031	13
Dataset_07_SARS-CoV-2_049	44
Dataset_07_SARS-CoV-2_049	45
Dataset_07_SARS-CoV-2_035	30
Dataset_07_SARS-CoV-2_058	32
Dataset_07_SARS-CoV-2_085	35
Dataset_09_SARS-CoV-2_101	1
Dataset_09_SARS-CoV-2_101	2
Dataset_10_SARS-CoV-2_012	3
Dataset_09_SARS-CoV-2_084	4
Dataset_09_SARS-CoV-2_076	5
Dataset_09_SARS-CoV-2_092	15
Dataset_02_SARS-CoV-2_089	5
Dataset_10_SARS-CoV-2_062	13
Dataset_10_SARS-CoV-2_062	15
Dataset_10_SARS-CoV-2_062	16
Dataset_10_SARS-CoV-2_062	17
Dataset_10_SARS-CoV-2_062	18
Dataset_10_SARS-CoV-2_062	19
Dataset_08_SARS-CoV-2_049	21
Dataset_08_SARS-CoV-2_049	23
Dataset_08_SARS-CoV-2_049	25
Dataset_08_SARS-CoV-2_029	1
Dataset_08_SARS-CoV-2_029	2
Dataset_08_SARS-CoV-2_006	11
Dataset_07_SARS-CoV-2_049	31
Dataset_07_SARS-CoV-2_058	33
Dataset_07_SARS-CoV-2_085	36
Dataset_07_SARS-CoV-2_086	37
Dataset_10_SARS-CoV-2_063	1
Dataset_09_SARS-CoV-2_029	17
Dataset_10_SARS-CoV-2_062	20
Dataset_09_SARS-CoV-2_076	6
Dataset_09_SARS-CoV-2_076	7
Dataset_09_SARS-CoV-2_007	8
Dataset_09_SARS-CoV-2_007	9
Dataset_09_SARS-CoV-2_007	10
Dataset_09_SARS-CoV-2_004	11
Dataset_09_SARS-CoV-2_005	12
Dataset_09_SARS-CoV-2_056	13
Dataset_09_SARS-CoV-2_056	14
Dataset_09_SARS-CoV-2_092	16
Dataset_09_SARS-CoV-2_029	18
Dataset_09_SARS-CoV-2_029	20
Dataset_08_SARS-CoV-2_049	24
Dataset_08_SARS-CoV-2_029	3
Dataset_08_SARS-CoV-2_029	4

Dataset_08_SARS-CoV-2_029	5
Dataset_08_SARS-CoV-2_024	7
Dataset_08_SARS-CoV-2_024	8
Dataset_08_SARS-CoV-2_006	10
Dataset_07_SARS-CoV-2_085	34
